# The Off-Patent Biological Market in Belgium: Is the Health System Creating a Hurdle to Fair Market Competition?

**DOI:** 10.3390/ph14040352

**Published:** 2021-04-10

**Authors:** Philippe Van Wilder

**Affiliations:** Ecole de Santé Publique, Université Libre de Bruxelles (ULB), Brussels 1050, Belgium; philippe.van.wilder@ulb.be

**Keywords:** biosimilar, reference biological, competitive market, Belgium

## Abstract

We investigated the off-patent biological market in Belgium from a policy maker’s perspective, in light of the Belgian pharmaceutical health system. The main barriers relate to a short-term budgetary focus, to the overwhelming innovator’s reach and to a concertation model with assessment and appraisal being mixed which results in poorly effective policy measures.

## 1. Introduction

Biosimilar products have been authorised in the European union in areas such as oncology, inflammatory diseases, diabetes and haematology. As these products are authorised as having no clinically meaningful differences [1] compared to the originator products, the major benefits relate to lower prices for the payer (health insurance), following lower development costs, and in enhanced price competition in theory. The savings may serve as a healthcare budget control tool or may be invested in widening the eligible patient group or in providing access to other innovative but expensive treatments.

Many EU member states have experienced a gradual uptake [2] of biosimilar products with uptake figures [3] in 2019, easily exceeding 30% in big EU-countries while in Belgium [4,5] the uptake is incredibly low with market shares of biosimilar products being three-times lower or less.

Different authors have analysed the limited uptake of biosimilar products in Belgium and identified several factors responsible for this phenomenon. Recently, Moorkens et al. [6] provided a critical overview of these hurdles which include a lack of trust among some stakeholders, a lack of clear, persistent and consistent communication channels, a tremendous innovator’s reach and the disturbing impact of a hospital financing system with incentives for high priced medicines.

In this commentary, we aim to consider the impact of the organisation of the pharmaceutical healthcare system. We believe the cumulative impact of distinct policy measures within the Belgian healthcare system is creating a strong hurdle to the access of biosimilar products.

## 2. Belgian Policy Initiatives to Favour Access to Biosimilar Products

Because of the low use of biosimilar products in Belgium, various policy measures aiming to impact the biosimilar uptake have been implemented. First, there is a simplified and facilitated administrative reimbursement track [7] for biosimilar products if the formal indications and conditions of use are nearly identical between biosimilar and reference biological product. In 2015, a Convention was signed by the competent authority and the main stakeholders [8], to enhance the biosimilar product uptake. This initiative included a regular monitoring of the use of biosimilar products to check this objective. In 2019, financial incentives [9] (between € 750 and € 1500) were provided towards individual prescribers of the biosimilar products of etanercept and adalimumab in the retail market. Finally, a ‘best value biological’ program management [10] was initiated in 2019, aiming to bring a broader perspective to the use of biological medicinal products [11].

After all of these ad hoc initiatives, the Belgian biosimilar market share evolution illustrates the lack of competition between biosimilar and biological reference product. Clearly, the biosimilar product uptake objectives are not reached at all despite these ad hoc measures.

The relationship between competent authority and pharmaceutical industry is complex and particular. In the BE Healthcare system, decision-making is based on extensive concertation with stakeholders. This allows to capture thoughts from the field and enables the decision-making to hold a balanced view of the expressed legitimate opinions, but it may lack strength because of the weighting of the impact across the stakeholders.

In my opinion, the biosimilar market uptake in Belgium suffers from a short-term narrow budgetary focus, the limits of a concertation model with mixed expert and stakeholder input which (in this field) result in poorly effective policy measures and from the overwhelming innovator’s reach.

## 3. Some Characteristics of the Pharmaceutical Healthcare System

### 3.1. Short-Term Budgetary Focus

The Belgian healthcare system emphasises the short-term (pharmaceutical) healthcare budget instead of considering the healthcare budget and the longer-term efficiency. To illustrate this, I would refer to the mandatory price decreases that are applicable 12 years after the reimbursement of the reference product or at entrance of the biosimilar product, whichever comes first. These price reductions are applicable to both the reference product and the biosimilar product. The consequence of this policy measure is that there is no or limited additional saving potential between a biosimilar or biological reference product for the healthcare payer. This effect is strengthened by the hospital financing mechanism in which a lower (biosimilar) price would result in less earnings for the hospital, because the discounts offered by a manufacturer are lower if the National Institute of Health and Disability Insurance (NIHDI) list price is lower.

In some cases, the potential for price competition is even further hampered by repeated managed entry agreements (MEAs) for the reference biological product. If such a confidential MEA is still effective at the entrance of the biosimilar product, the type of agreement and the extent of the price–volume compensation is unknown to the applicant of the biosimilar product, making the biosimilar reimbursement submission a blind spot.

Price decreases go beyond 50% resulting in much desired short-term cost savings but without incentives to increase the market share of biosimilar products with the aim to facilitate longer-term competition once biosimilar products achieve substantial market volumes. A mix of off-patent reference biologics, biosimilars, and follow-up treatment alternatives, is considered critical [12] to obtain a competitive and sustainable market for long term access to biological therapy at the lowest cost. The actual regulation is applicable even if the NIHDI is aware of the risks linked to a lack of competition. On its website [13], it expresses the concern of missing future biosimilar products on the Belgian market, which may be a threat to the sustainability and control of the healthcare budget.

The already mentioned simplified reimbursement track for biosimilar products is limited to 90 days (which is half the duration of other reimbursement procedures). It builds on the similarity in the clinical and therapeutic characteristics between biological reference and biosimilar product and will mainly address the budget impact estimates, without any pharmaco-economic assessment. Cost-effectiveness analyses may be of little value in some cases but they are indicated [14] if cost differences exist between these products (administration route and costs, dosing interval, limited price differences, etc.) and if therapeutic alternatives exist on a different anatomic-therapeutic-chemical (ATC)-level, a pharmaco-economic assessment will probably result in a different relative positioning of the available products. The focus should be more directed towards disease strategy and look beyond individual medicinal products.

### 3.2. Mixing Assessment and Appraisal

The biosimilar market uptake is also affected by the unclear split between assessment and appraisal in the healthcare decision-making. This also affects other medicinal products. The Belgian reimbursement procedure reflects the Transparency Directive [15] requirements and starts with an internal assessment report to be delivered by day 60, followed by appraisal and proposition from the reimbursement commission by day 120 and day 150, prior to the decision of the Minister. However, in my opinion, despite the tremendous support of a dedicated team of NIHDI-experts and pharmacists, assessment and appraisal are sometimes mixed [16] instead of these being clear and distinct activities. This is mainly the consequence of having a commission of stakeholders [17] all along the procedure instead of having distinct expert assessors and stakeholder appraisers. This approach is hampering the scope of the performed assessments, resulting, too often, in limited pharmaco-economic assessments. It is limited because it is not going beyond the individual submitted dossier and because it merely criticises the applicant’s submission without making own assumptions and performing own economic analyses. Until now, the impact of the economic assessment as a significant reimbursement factor, could not be demonstrated as opposed to the significant effect of the medical need and the extent of therapeutic value.

The biosimilar conundrum is a topic in which the pharmaco-economic assessment, even from a direct healthcare perspective, should go beyond the individual dossier and include elements of (lack of) future competition (especially if a lifelong time horizon is relevant). A proposal might be to strengthen the possible collaboration between the NIHDI and the formal health technology assessment (HTA) Agency which is the Federal Knowledge Centre for health (KCE). KCE has the expertise for making full HTA-reports. Assessment reports should be ‘validated’ by expert assessors from NIHDI and KCE and remain unchanged by the reimbursement commission of stakeholders. The Commission members should focus on the appraisal of the assessment report and discuss criteria and weights to provide a documented and informed advice to the competent Minister. Such collaboration would facilitate assessments that go ‘beyond the dossier’ and include longer-term considerations. Such collaboration may enable the reimbursement commission to prepare for future challenges and develop an adapted framework for oncology biological products.

### 3.3. Far Reaching Innovator’s Reach

The biosimilar market uptake is also affected by the originator’s extensive reach [6] and creative off-patent strategy. The innovative pharmaceutical industry is strongly represented in Belgium with R&D centers and/or production facilities of companies such as Glaxo-Smith Kline (GSK), Johnson & Johnson, or Pfizer. GSK’s site in Rixensart produces various vaccines for world-wide distribution. Pfizer manufactures in Puurs the COVID-19 vaccine Comirnaty^®^ which is distributed across the EU and abroad. The innovative industry [18] represents an export volume of more than € 42 billion in 2018 and invested € 3.6 billion in the development of medicinal products in 2018 (R&D is facilitated by fiscal incentives).

The COVID-19 pandemic offers a marketing opportunity, certainly for the vaccine producing pharma, but also for the whole pharmaceutical sector, if the sector delivers highly valued medicinal products protecting individuals from disease and excessive mortality and making it possible for societies to reopen economic and societal activities.

This innovative industry is particularly creative to manage the off-patent hurdle. Follow-up compounds with claims of added value enter the market prior to the patent expiry and are being promoted to facilitate a shift from the biological product to the follow-up product (e.g., Janus kinase (JAK) inhibitors versus tumor necrosis factor (TNF) blockers).

The biosimilar product manufacturers should consider their own strengths and weaknesses in this ‘originator’ landscape. They should not only be reactive to the activities of the originator but also develop proactively own strategies, expressing their commitment towards local authorities and stakeholders (including patients), reflecting on product differentiation and investing in a tangible roadmap to achieve the objectives.

The originator is also rapidly reactive to the changing market situation. Initially, as the analysis of Dutch market data [19] illustrates, substantial price differences existed between adalimumab reference product and biosimilar products, which resulted in decreasing market shares for the adalimumab reference product. For etanercept, no or limited price differences exist and market shares of the biological reference product did not dramatically decrease.

A collateral ‘benefit’ of this tactical approach, is that originator and biosimilar products provide a similar amount of savings, which allows the originator industry to make the (legitimate) claim of facilitating the pharmaceutical healthcare budget control (which budget had an excessive double-digit growth in 2020).

## 4. The ‘Best Value Biologicals’ Program 2019–2020

With a less than desirable interest in longer term efficiency in the Belgian healthcare system, the claim by the originator that savings are equally resulting from reference and biosimilar products, yields a considerable threat to any future incentive for biosimilar products.

To illustrate this, the example of the 2019 ‘best value biologicals’ program management is appealing.

The ‘best value biologicals’ program management, sponsored by the NIHDI and which may be considered as the initiation of a broader and sustained approach towards biological products, ended in April 2020. The program delivered to the competent authority NIHDI, a documented report including recommendations with suggested follow-up measures aiming to create a level playing field for biosimilar and biological reference products in Belgium. Briefly, the report’s recommendations referred, among others, to extensive and repeated communication and education channels on biological products to healthcare providers and patients, to smoothen the lengthy and complex tendering process in hospitals, to consider gain sharing mechanisms (sharing the healthcare savings with those enabling them) and a specific recommendation on setting market share quota for biosimilar products.

However, the coronavirus pandemic interfered strongly and obviously modified healthcare priorities.

Still, the report’s recommendations were endorsed later, in October 2020, by the General Council of the NIHDI [20] in their approval of the 2021 federal healthcare budget. However, the wording used by the General Council was particular: when it comes to the intention to stimulate the use of biosimilar products, a condition is set and the wording ends with ‘with non-discriminatory incentives’.

The General Council inserted the ‘no discrimination’ condition, prior to any concrete policy measure being written. For initiatives relating to biosimilar product uptake, an a priori condition is already ‘adopted’, prior to making clear how the policy measure should be conceived and implemented. No other healthcare initiative in the text of the General Council is linked to a non-discriminatory criterion.

Maybe, the proposal to use quota for uptake of biosimilar products is perceived as ‘discriminatory’ by some General Council members. However, quota is used by other EU Member States. At the EC-workshop of October 2019 on biosimilar products [21], various Member States shared their experience on the effectiveness of local policy measures. France [22] explained their positive pilot experience with quota for biosimilar products which resulted in increased biosimilar market shares. This enabled them to reach a competitive level playing field between original and biosimilar biological products. The French experience was acceptable for the EC, because the economic rationale was clear and unambiguous and the quota policy was limited in time under strict conditions.

## 5. Conclusions

By having the ‘with no discriminatory incentives’ written in the General Council document, any forthcoming policy measure creating incentives for the uptake of biosimilar products will be challenged on this condition. The perception of discrimination is made and any initiative having a purpose to enhance uptake of biosimilar products will not only need to be valid for that purpose, but also reverse the created perception. The formulation of the various recommendations and the implementation of concrete measures will probably be an uphill battle, with opportunity for the originators to have lengthy discussions on the compliance of each measure with the non-discrimination condition. In my understanding, some of the proposed recommendations giving incentives to biosimilar products may be differential but not discriminatory, because they are limited in time and are proposed to create a level playing field for both type of biological products, which is hardly the case at patent expiry of the reference biological product. It will be important to refer to the international examples to reverse the perception of discrimination already linked to upcoming incentives.

At stake are the availability of the next biosimilar products in Belgium and the shift towards a real competitive market. The expected future savings in oncology and rare diseases, to be triggered by the local availability of biosimilar products, may be reduced and/or delayed. Non-differential mandatory price decreases do and will not resolve the conundrum of the biosimilar market uptake. Policy makers in Belgium should focus on the longer-term health economic assessment and strengthen the economic assessment capacity and scope (i.e., going broader and beyond the individual dossier). This is much needed to achieve a level playing field for biosimilar products in Belgium, 15 years after the first introduction in the EU.

## Data Availability

Not applicable.
